# Dynamically Modulated Core–Shell Microfibers to Study the Effect of Depth Sensing of Matrix Stiffness on Stem Cell Fate

**DOI:** 10.1021/acsami.1c06752

**Published:** 2021-08-06

**Authors:** Dan Wei, Laura Charlton, Andrew Glidle, Nan Qi, Phillip S. Dobson, Matthew John Dalby, Hongsong Fan, Huabing Yin

**Affiliations:** † National Engineering Research Center for Biomaterials, College of Biomedical Engineering, Sichuan University, Chengdu 610064, Sichuan, China; ‡ James Watt School of Engineering, University of Glasgow, Glasgow G12 8LT, U.K.; § Institute of Marine Science and Technology, Shandong University, Qingdao 266237, China; ∥ Centre for the Cellular Microenvironment, Institute of Molecular, Cell and Systems Biology, College of Medical, Veterinary and Life Sciences, University of Glasgow, Glasgow G12 8QQ, U.K.

**Keywords:** ECM, dynamic mechanics, depth sensing, effective modulus, osteogenesis differentiation

## Abstract

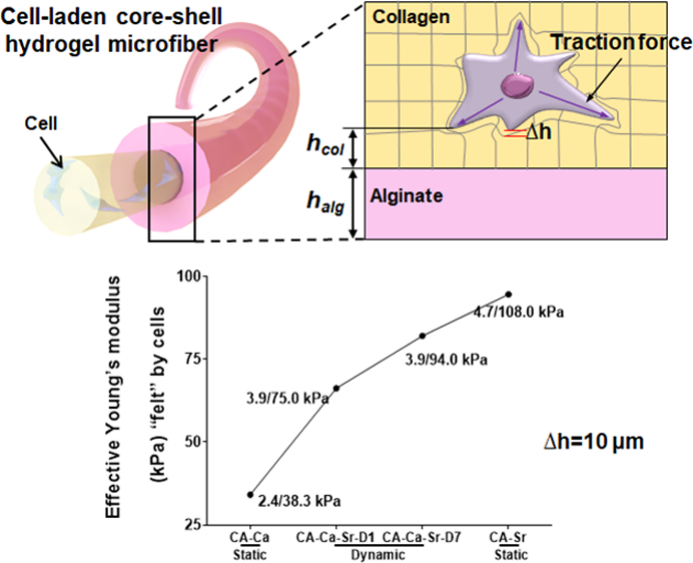

It is well known that extracellular matrix stiffness can affect cell fate and change dynamically during many biological processes. Existing experimental means for in situ matrix stiffness modulation often alters its structure, which could induce additional undesirable effects on cells. Inspired by the phenomenon of depth sensing by cells, we introduce here core–shell microfibers with a thin collagen core for cell growth and an alginate shell that can be dynamically stiffened to deliver mechanical stimuli. This allows for the maintenance of biochemical properties and structure of the surrounding microenvironment, while dynamically modulating the effective modulus “felt” by cells. We show that simple addition of Sr^2+^ in media can easily increase the stiffness of initially Ca^2+^ cross-linked alginate shells. Thus, despite the low stiffness of collagen cores (<5 kPa), the effective modulus of the matrix “felt” by cells are substantially higher, which promotes osteogenesis differentiation of human mesenchymal stem cells. We show this effect is more prominent in the stiffening microfiber compared to a static microfiber control. This approach provides a versatile platform to independently and dynamically modulate cellular microenvironments with desirable biochemical, physical, and mechanical stimuli without an unintended interplay of effects, facilitating investigations of a wide range of dynamic cellular processes.

## 1 Introduction

The natural extracellular matrix (ECM) microenvironment provides an evolving mechanical^1,2^ support to cells. Understanding the cellular response to dynamic mechanical cues in their microenvironment can reveal the key underlying mechanisms during tissue development or pathological processes. However, up to now, bidirectional mechanical interaction between the cell and matrix remains elusive, especially within a three-dimensional (3D) cell culture. To better explore the dynamic interactions between cells and their matrix, multiple stimulation strategies, such as the use of ultraviolet light,^3^ visible light,^4^ pH,^5^ temperature,^6^ and ion exchange,^7^ have been deployed to modulate the matrix’s mechanical properties at user-defined time points. So far, these reported methods share the common principle of resorting to varying the degree of cross-linking or controlling the density of hydrogels. These changes are known to affect cell adhesion and function, which could cause contradictory conclusions when studying the effects of matrix stiffness.^8,9^ Furthermore, reports studying cell behavior in 3D matrixes with a high stiffness (>100 kPa) are limited due to restricted cell growth and migration. Thus, it remains a significant challenge to establish a suitable platform to investigate how cell behaviors are influenced by wide variations in the mechanical properties of the matrix.

Intracellular traction forces generated by cells resisting the ECM via cytoskeleton contractility direct a number of cellular processes, including cell spreading, migration, and differentiation.^10,11^ The intracellular traction force, which is strongly related to ECM mechanical properties, can be transmitted onto/into the ECM via focal adhesions, subsequently leading to matrix displacement;^12,13^ the degree of displacement depends on matrix stiffness. In turn, this matrix displacement can trigger the movement of focal adhesions which then participates in the generation of an intracellular traction force via mechanically sensitive ligand-receptor pairs;^14,15^ the relationship between intracellular traction force and matrix stiffness is bidirectional.

Intracellular traction force, as regulated by cell–matrix interactions, consists of both parallel and vertical components. To date, the majority of studies only focus on the shear (parallel) traction, which is determined by the stiffness of the matrix that a cell is in direct contact with.^13^ However, they ignore the vertical traction force that is affected by the matrix’s thickness. Recently, some studies have reported that cells can not only feel the stiffness of a matrix but can also sense the thickness of it, which is important for generation of vertical traction forces.^16,17^ For example, Sen et al.^16^ found that cells seeded on a 2D thin soft hydrogel that was below a critical stiffness of 8 kPa began to sense the underlying rigid substrate, eventually generating higher intracellular stress compared to those on thick hydrogels of the same stiffness. It was also found that cell surface displacements decay exponentially in a stiffness independent manner over a characteristic length of 10 μm.^18^ Similarly, cells in a 3D matrix were observed to exert both parallel and vertical traction forces against the surrounding matrix^13,19^ with cell-generated forces causing ∼10 μm displacement of a collagen matrix around cells.^20^ Due to the close correlation between intracellular traction force and matrix-related mechanical cues, the effective stiffness probed by a cell is affected not only by the stiffness of the matrix which is directly in contact with the cell (parallel traction force) but also by the stiffness of an underlying substrate when the location of the cell is not far from the substrate, for example, within the critical depth of cell-sensing (vertical traction force). However, there is limited knowledge and no reports on the effects on cell fate of depth sensing.

Inspired by this concept of depth sensing by cells, we conceived an innovative ECM model with a hierarchical structure and composition, that is, a cell-laden core–shell microfiber, which can deliver dynamically modulated effective stiffness to cells without the need of modifying cells’ immediate ECM. It is worth highlighting that this is distinctly different from the reported methods so far, where in situ modulation of the matrix stiffness always involves physical and/or chemical changes to the matrix (as discussed above). In this model, cells are encapsulated in the core, which can be made of highly bioactive, soft natural ECM materials (e.g., collagen, matrigel). The shell is made of synthetic biomaterials to allow for easy modulation of its stiffness. To achieve this, microfluidic technology was employed as the method of choice, thanks to its ability to create desirable architectures with microscale resolution.^21−23^ We have recently established a microfluidic extrusion approach to create a range of cell-laden microfibers with well-controlled thickness and different compositions for individual layers.^22^ This platform therefore provides a versatile means to create an innovative ECM model.

**Figure 1:**
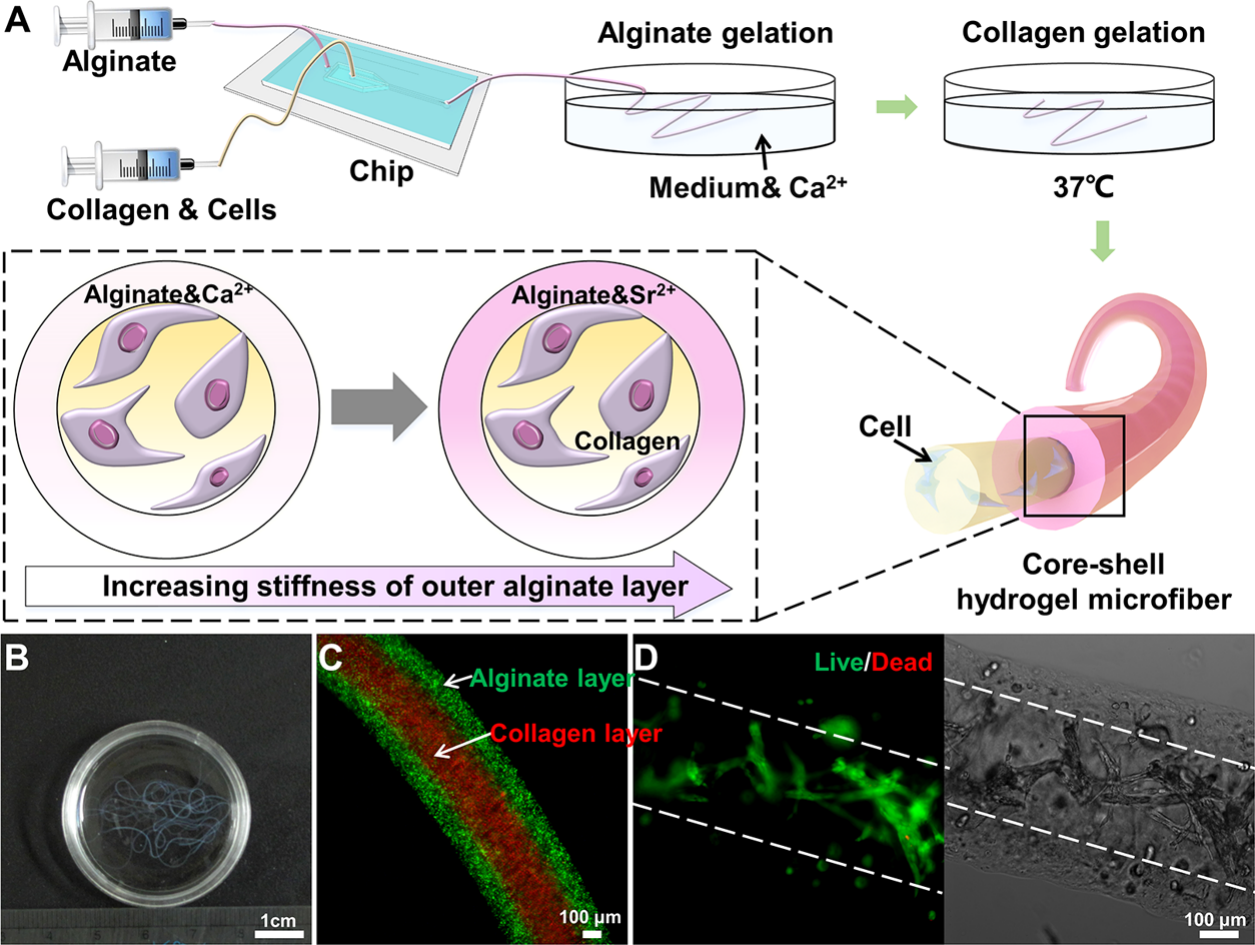
(A) Schematic drawing of continuous extrusion of core–shell microfibers and the principle of dynamic modulation of the mechanical properties of the outer alginate shell via ion exchange. (B) Photograph of continuous core–shell hydrogel microfibers. (C) Representative fluorescence image of the core–shell hydrogel microfibers with added red (collagen layer) and green fluorescence beads (alginate layer) illustrating individual layers. (D) Fluorescence image (left) and bright field image (right) of MG63s encapsulated in collagen core after 7 days in culture.

For this first study, we created simple, dynamically stiffening, cell-laden microfibers with a collagen core layer and an alginate shell layer (Figure 1A). Alginate solutions gel instantly in the presence of divalent cations, such as calcium (Ca^2+^) and strontium (Sr^2+^). However, strontium has a higher binding affinity to alginate than calcium, creating a stiffer alginate gel.^24^ This difference was utilized to create dynamically stiffening microfibers for the cell studies. Atomic force microscopy (AFM) was used to characterize the cross-section modulus of the dynamically stiffening core–shell microfiber over time. The effective stiffness sensed by cells was also calculated using finite element (FE) simulation. Human mesenchymal stem cells (hMSCs) were used as a cell model with a dynamic phenotype. In response to our novel materials, we observed drastically different behaviors (e.g., spreading, proliferation and differentiation) when they were exposed to mechanical cues (i.e. from the microfibers) compared to that without (i.e., cells in bulk).

## 2 Results and Discussion

### 2.1 Fabrication of Cell-Laden Core–Shell Microfibers

We employed microfluidic extrusion to construct the core–shell microfiber with a thin collagen core to provide a bioactive microenvironment for cell growth, and an alginate shell with ionic-controllable stiffness to deliver distantly modulated mechanical cues during culture (Figure 1A). The continuous microfluidic extrusion allowed for meters-long microfibers to be easily obtained (Figure 1B). To aid visualization, red and green fluorescence beads were added to the collagen and alginate solutions, respectively. The diameter of whole prepared microfiber was 498.8 ± 4.4 μm, and the diameter of the collagen core part was 284.4 ± 5.4 μm (Figure 1C).

Human osteoblast-like cells (MG63s) were initially used to create cell-laden microfibers. As shown in Figure 1D, the vast majority of the MG63s were well spread and remained alive and were distributed across the whole core area after 7 days in culture, suggesting that the fabricated collagen core provides an excellent biocompatible microenvironment for cell adhesion and viability.

### 2.2 Mechanical Characterization of the Whole Microfibers

The microfiber stiffness was dynamically modulated due to the cross-linking of alginate via divalent ions being a reversible process. Therefore, ions in the alginate shell can diffuse out or can be replaced by other divalent ions depending on the electrolytes in the culture medium. Because Sr^2+^ has a higher affinity to alginate than Ca^2+^, SrCl_2_ was added into the culture media to gradually replace Ca^2+^ ions in the alginate shell and thus produce a stiffer shell. The microfibers created in this way were denoted as CA–Ca–Sr fibers (stiffening fibers). The microfibers formed in CaCl_2_ solution and supplemented with CaCl_2_ were named CA–Ca fibers (soft fibers), and those formed in SrCl_2_ solution and supplemented with SrCl_2_ were named CA–Sr fibers (stiff fibers). Pure alginate (PA) microfibers and pure collagen (PC) microfibers were formed as controls.

**Figure 2:**
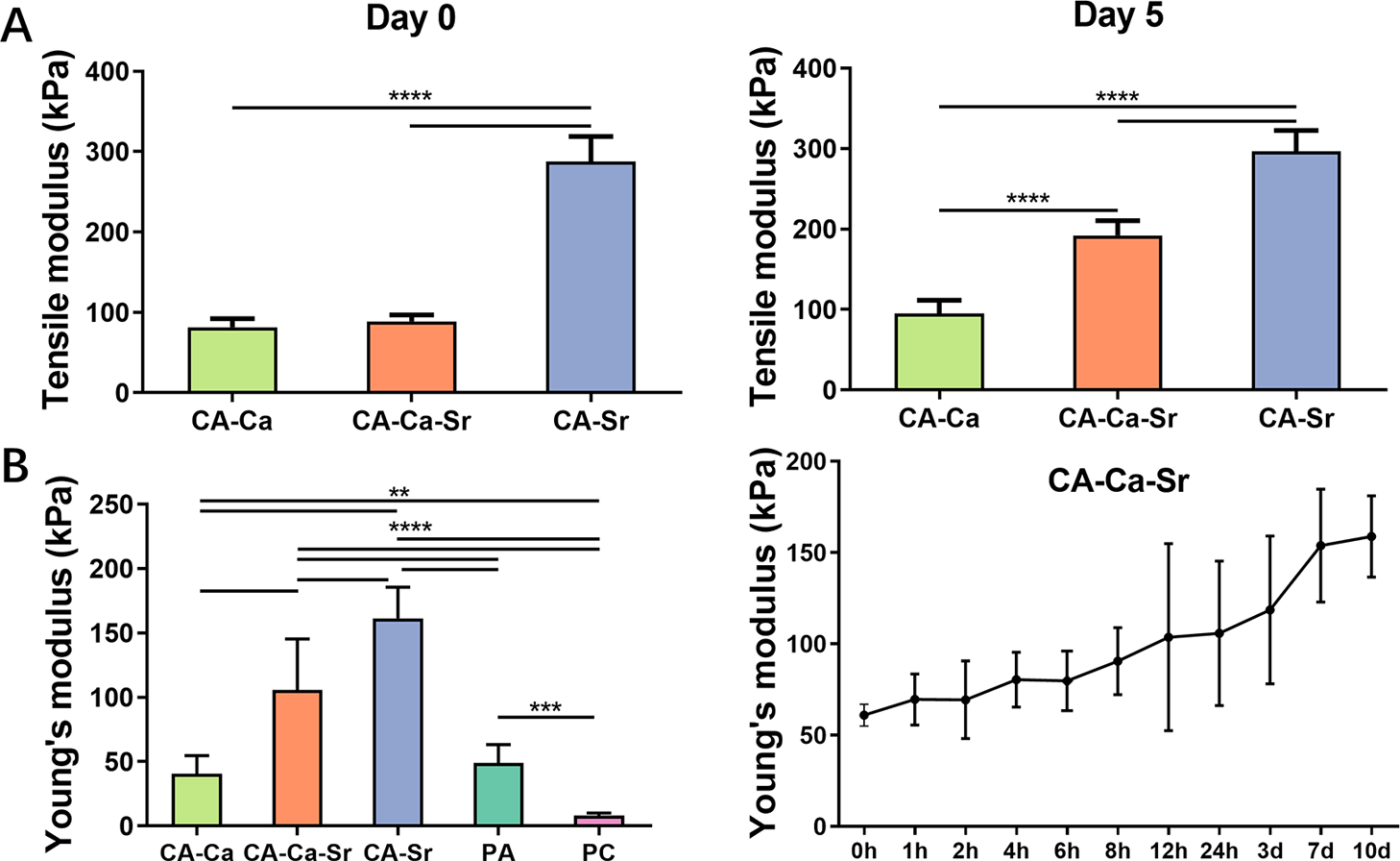
Mechanical testing of the microfibers over time. (A) Tensile modulus of the whole fibers (*n* = 5). The three types of microfibers were kept in 100 mM CaCl_2_ or SrCl_2_ supplemented medium for 5 days. (B) Young’s modulus of the microfibers at day 1 (left) and that of CA–Ca–Sr fibers kept in 100 mM SrCl_2_ supplemented media for 10 days (right). Young’s modulus was determined by AFM nanoindentation and thus mainly reflects the stiffness of the alginate shell (*n* = 12). CA–Ca = fibers set and maintained in Ca^2+^ solution; CA–Ca–Sr = fibers set in Ca^2+^ solution and maintained in Sr^2+^ solution; CA–Sr = fibers set and maintained in Sr^2+^ solution; PA = pure alginate; PC = pure collagen. **P < 0.01, ***P < 0.001, ****P < 0.0001.

To measure the mechanical properties of the whole microfibers, a combination of tensile micro-testing and AFM indentation was used. At day 0, the initial tensile modulus of CA–Sr microfibers (288.0 ± 35.9 kPa) was almost three times higher than that of CA–Ca fibers (78.2 ± 15.3 kPa) and CA–Ca–Sr fibers (86.9 ± 13.0 kPa) (Figure 2A). These results agree well with the previous work, which shows strontium ions leading to the formation of stronger alginate gels compared with other divalent ions.^24^ After 5 days in supplemented media, the tensile modulus of both CA–Sr and CA–Ca static fibers remained constant. However, there was an almost 2-fold increase in the tensile modulus of CA–Ca–Sr stiffening microfibers (190.6 ± 22.5 kPa), showing that the replacement of Ca^2+^ in the alginate gel by Sr^2+^ ions from the supplement media results in increased stiffness.

The compressive Young’s modulus of CA–Ca, CA–Ca–Sr, and CA–Sr fibers after 24 h in media containing their respective cations as well as PA and PC control fibers were determined using AFM (Figure 2B, left). As expected, the stiffness of PC was significantly lower than the other microfibers. No significant difference was found between the CA–Ca and the pure PA control because the alginate shells of both fibers were set in the same Ca^2+^ concentration. Similar to the results of the tensile strength testing, CA–Sr fibers had the highest Young’s modulus (162.0 ± 24.5 kPa), while the CA–Ca–Sr stiffening fibers (106.0 ± 40.7 kPa) were significantly stiffer than the CA–Ca fibers (39.9 ± 15.3 kPa). From a series of time course experiments on the CA–Ca–Sr fibers in media containing 100 mM SrCl_2_, it was found that their average stiffness increased consistently from 61.0 ± 5.9 to 158.7 ± 22.2 kPa over 10 days, gradually plateauing after 7 days (Figure 2B, right). These results confirmed successful dynamic manipulation of mechanical properties of the microfibers via ion exchange.

Considering the possible effects of a high concentration of divalent ions on cell viability, cell-laden microfibers with MG63s were cultured in growth media containing a range of CaCl_2_ or SrCl_2_ concentrations (Figure S1). Live/dead viability staining results after 1 day of culture showed a gradual reduction in cell viability with increased cation concentration. However, cell viability was >80% when CaCl_2_ and SrCl_2_ were used between 2.5 and 10 mM. Hence, a 10 mM concentration of CaCl_2_ or SrCl_2_ was used for further experiments with cell-laden microfibers.

### 2.3 Mechanical Characterization of the Cross Section of the Microfibers

**Figure 3:**
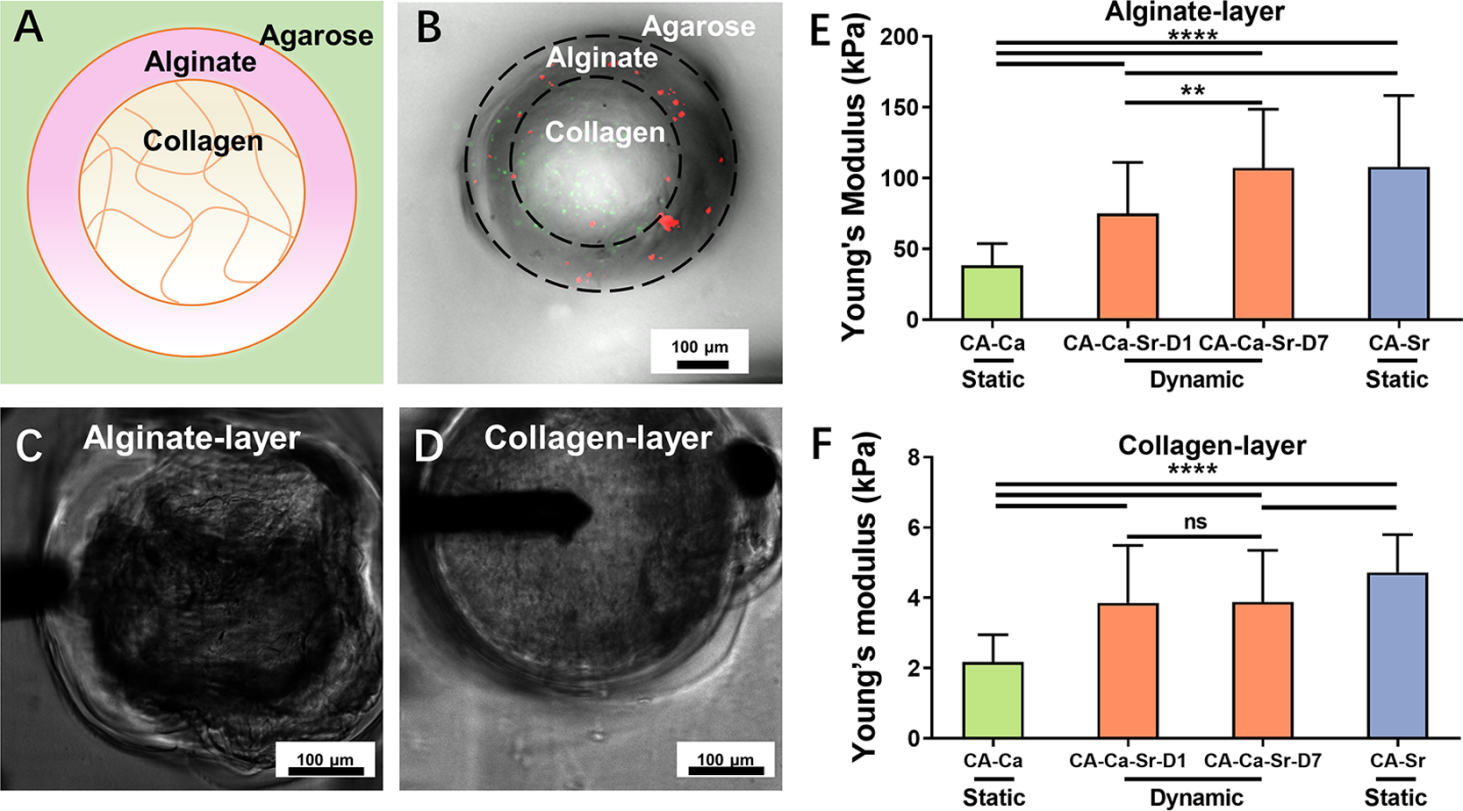
Modulus of different layers of core–shell microfiber. (A) Schematic diagram of the cross section of a microfiber embedded in agarose gel. (B) Combined bright-field and fluorescence image of the cross-section of a two-layered fiber with added red (alginate) and green (collagen) beads. Phase image of the alginate layer (C) and collagen layer (D) of core–shell fiber during AFM indentation (note: the black lever is an AFM probe). Graph of Young’s modulus of the alginate shell layer (E) and collagen layer (F) of the core–shell fibers. D1 and D7 indicate the fibers that were kept in 10 mM CaCl_2_- or SrCl_2_-supplemented media for 1 day or 7 days. (*n* ≥ 60), **P < 0.01, ****P < 0.0001.

To investigate the stiffness changes in the alginate shell layer and monitor whether the collagen core was affected by the cations present in the media during long-term culture, the microfibers were embedded in agarose gel and cross-sectioned (Figure 3A). This method facilitated the handling of individual fibers, revealing a clear boundary (Figure 3B). Young’s modulus of the core and shell layer was measured using AFM indentation overlaid with phase images of the microfiber’s cross-section (Figure 3C,D). On day 1, the cross-section results of alginate shells follow the same trend in Young’s modulus as those of the whole fibers in Figure 2, that is, CA–Ca (38.3 ± 15.4 kPa) significant change was found for the alginate shell of the CA–Ca fibers after 7 days in Ca^2+^-supplemented media compared with that at day 1 (data not shown). However, after 7 days in Sr^2+^-containing media, the alginate shell of the CA–Ca–Sr stiffening fibers (CA–Ca–Sr–D7) had stiffened significantly to 94.2 ± 37.3 kPa, approaching to the value of CA–Sr fibers (Figure 3E).

In contrast, the stiffness of the collagen core in the CA–Ca–Sr fibers showed no significant difference over time (Figure 3F), with a Young’s modulus of 3.8 ± 1.6 kPa at day 1 and of 3.8 ± 1.5 kPa at day 7. This suggests that the exchanged ions did not affect the structure of the collagen hydrogel. The collagen core of the CA–Ca fibers had a Young’s modulus of 2.4 ± 1.1 kPa and that of CA–Sr fibers had a higher Young’s modulus of 4.7 ± 1.1 kPa. This was likely caused by Sr^2+^ in the initial gelation solution, resulting in a slightly denser structure. Overall, the stiffness of the collagen core was <5 kPa; such a low stiffness for a bulk gel would predominantly promote differentiation to soft tissue phenotypes from the MSCs, such as adipocytes.^25^

### 2.4 Calculation of Effective Young’s Modulus Probed by Cells

**Figure 4:**
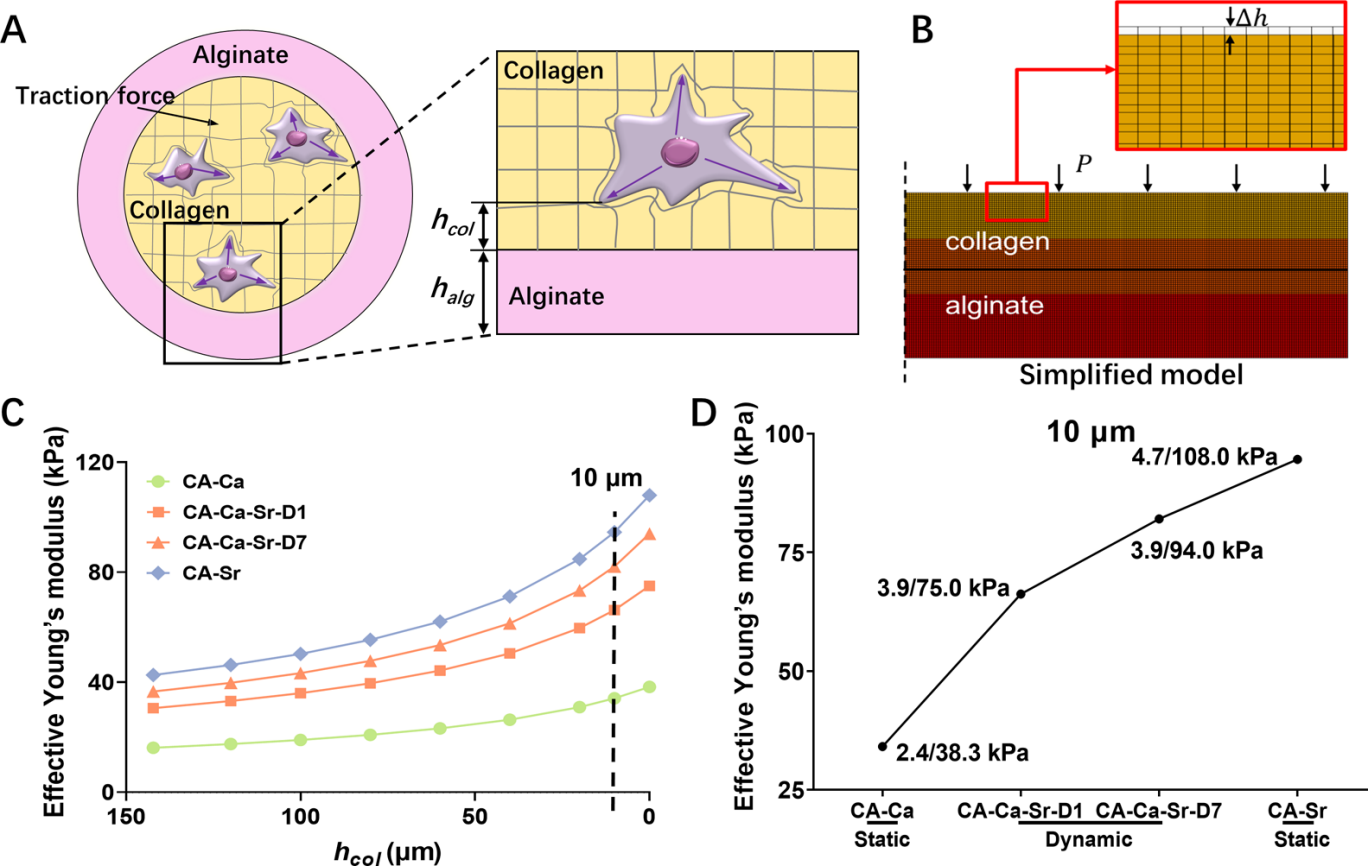
Effective stiffness across the collagen core. (A) Cells sense the surrounding matrix stiffness and generate traction forces, causing matrix displacement. *h*_col_ is the distance from the core–shell interface (i.e., *h*_col_ = 0) to the point where a cell exerts force (P). *h*_alg_ is thickness of alginate layer. (B) FE simulation of the effective stiffness of core–shell fibers. Δ*h* is the displacement from the upper surface between cell and matrix. (C) Simulation results of the effective matrix modulus across the collagen core. (D) Representative effective Young’s modulus “felt” by cells at *h*_col_ = 10 μm. Parameters for FE simulation: *h*_col_ varies from at the core–shell boundary (i.e., 0 μm) to the center of the core (i.e., 142.2 μm—the radius). *h*_alg_ = 107.2 μm. Young’s modulus of the collagen core and alginate shell from the cross-section indentation were used in the simulation, which are shown as *E*_col_/*E*_alg_ (e.g., 2.4 kPa/38.3 kPa) in (D).

As described above, if cells are cultured on a thin double-layered substrate, the effective stiffness “felt” by a cell, is influenced by the stiffness and thickness of its surrounding matrix as well as the underlying substrate’s stiffness.^13^ For the cell-laden core–shell microfibers, collagen was the immediate matrix surrounding a cell, and the alginate shell was the underlying substrate that provided indirect mechanical cues. As illustrated in Figure 1D, cells spread over the whole collagen core, with many cells having a part of their body in close proximity to, or even entering, the boundary of the alginate shell. Cell-generated intracellular traction forces could generate displacement in the surrounding collagen matrix, which in turn had an impact on intracellular traction force (Figure 4A). Considering the low stiffness (<5 kPa) and thickness of the collagen cores (radius = 142.2 ± 2.7 μm), cells would sense the outer alginate shell, in particular, for those located near the core–shell boundary.^16^ Thus, the effective stiffness of the collagen matrix that cells felt should include a contribution from the alginate shell.

To calculate the effective stiffness along the radius of the collagen core, a FE (finite element) simulation was implemented in Abaqus 6.14-4. Considering the concentric structure of the core–shell microfiber, an axisymmetric model for collagen–alginate double-layer with different heights *h*_col_ + *h*_alg_ and loading pressure *P* (numerical simulation of intracellular traction force) was put forward (Figure 4B). The effective Young’s modulus (*E̅*) that a cell at a certain position *h*_col_ in collagen could sense is expressed by the following formula.
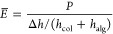
1where Δ*h* (caused by matrix deformation) is the displacement under loading *P* at the upper surface. *h*_col_ is the distance between a cell and the collagen–alginate interface. *h*_alg_ is the thickness of the alginate layer. *P* is the intracellular traction force. It is worth noting that the effective modulus is propositional to *P*/Δ*h*, but independent of *P* itself as the material was assumed linear, so the specific value of *P* does not change the value of *E̅*.

Figure 4C illustrates the effective Young’s modulus *E̅* as a function of *h*_col_, where *E̅* increases from the center point of the collagen core (i.e., *h*_col_ = 142.2 μm) to the core–shell interface (i.e., *h*_col_ = 0 μm). The gradient is steeper if the alginate’s stiffness is larger, or the *h*_col_ is small. Take *E̅* at the *h*_col_ of 10 μm as an example (Figure 4D). It is 34.1 kPa for the static CA–Ca fiber (*E*_col_/*E*_alg_ = 2.4 kPa/38.3 kPa) and 94.6 kPa for the CA–Sr fiber (*E*_col_/*E*_alg_ = 4.7 kPa/108.0 kPa). For the dynamic CA–Ca–Sr fiber, the *E̅* increased from 66.3 kPa at day 1 to 82.1 kPa at day 7 due to the increased alginate layer’s stiffness over time. These results indicate that the effective Young’s modulus “felt” by cells within the collagen core was significantly higher than the measured collagen stiffness and was dependent on the stiffness of the alginate shell.

### 2.5 Cellular Behavior of hMSCs in Response to Dynamically Tuned Young’s Modulus

#### 2.5.1 Proliferation and Morphological Response

Cell growth and morphology in the microfibers were monitored over time (Figures S2 and 5A). Both MG63 cells and hMSCs showed excellent viability. Previous studies showed that cells can spread in bulk 3D hydrogels with low stiffness (∼5 kPa) but are rounded in stiffer gels (∼20 kPa).^18^ Here, although the collagen cores of the three fibers were less than 5 kPa, spreading cells were mainly found in the center of the collagen core, while cells near the collagen–alginate boundary were rounded as if they were in a stiff gel (Figure S2). This agrees with the calculated effective Young’s modulus near the boundary (e.g., *E̅* >30 kPa at *h*_col_ of 20 μm), indicating depth sensing of the stiff alginate shell by cells. The different morphologies of cells within the first 4 days in culture clearly illustrated this phenomenon: the effective stiffness of the CA–Ca core was lowest, which saw many spread and elongated cells; the effective stiffness of the CA–Ca–Sr core in day 1 was relatively low, which resulted in some spreading cells; in contrast, very few cells spread in the CA–Sr core which had the highest effective stiffness (>60 kPa).

**Figure 5:**
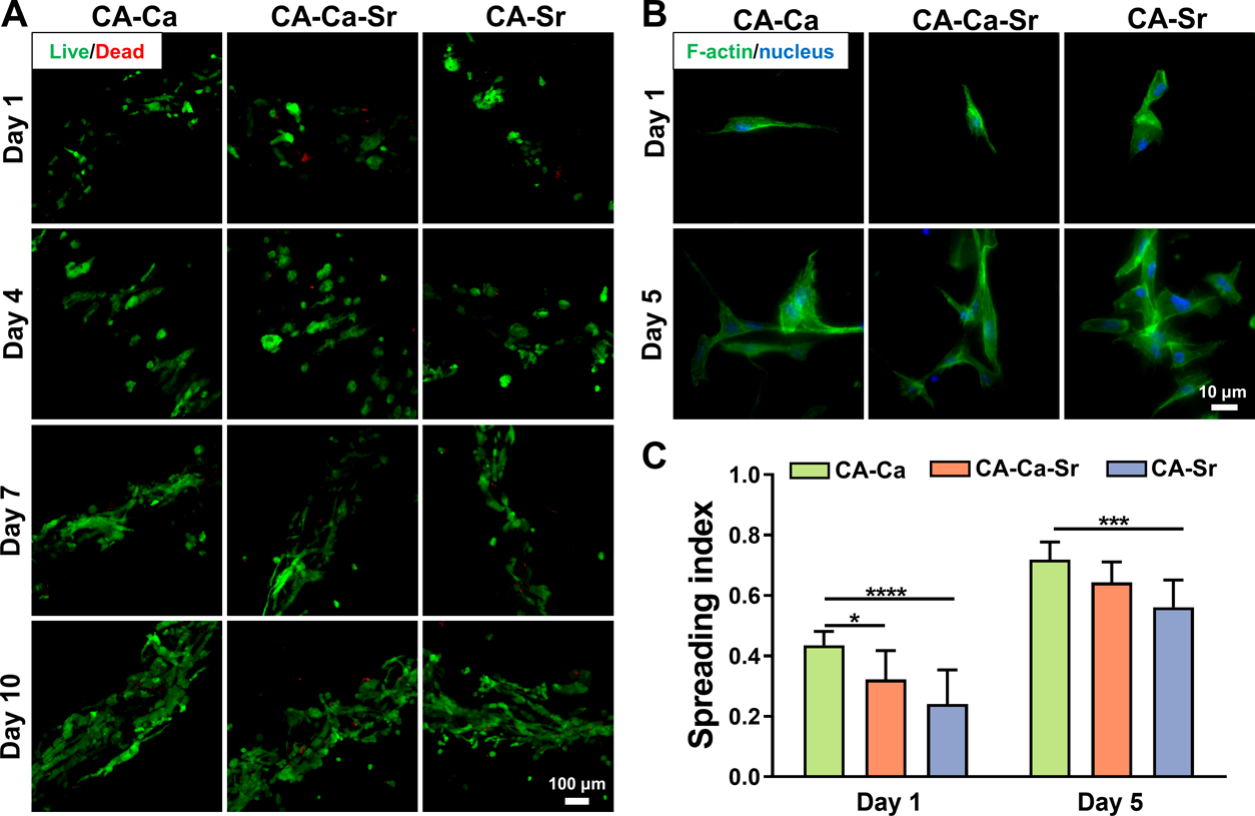
Morphological response of hMSCs in the collagen cores within different microfibers. (A) Fluorescent images of live (Calcein-AM, green) and dead (PI, red) stained hMSCs cultured in three microfiber groups for 1, 4, 7 and 10 days. (B) DAPI (blue) and F-actin staining (green) of hMSCs cultured in three microfiber groups after 1 day and 5 days. (C) Quantification of hMSCs spreading using the spreading index (detailed in the Experimental Section and Figure S3. Generally, a perfectly round cell has a spreading index of 0, while an elongated cell has a spreading index approaching 1). (cell number, *n* > 50), *P < 0.05, ***P < 0.001, ****P < 0.0001.

With extended time in culture, hMSCs in the three fibers all showed obvious spreading morphology (Figure 5A,B). However, hMSCs in CA–Ca fibers elongated and spread more than those in CA–Ca–Sr and CA–Sr fibers. The spreading index of the cells in the three fibers followed the trend: CA–Ca > CA–Ca–Sr > CA–Sr (Figures 5C and S3) throughout the culture period. At day 1, the spreading index was 0.44 ± 0.05 for CA–Ca, 0.32 ± 0.10 for CA–Ca–Sr, and 0.02 ± 0.11 for CA–Sr, respectively. At day 5, the spreading index was 0.72 ± 0.06 for CA–Ca, 0.64 ± 0.07 for CA–Ca–Sr, and 0.56 ± 0.09 for CA–Sr, respectively. At the end of day 10, the mean values of cell confluence for hMSCs were ∼28, 19, and 14% for the soft, dynamically stiffening, and stiff microfibers, respectively (Figure S4), indicating the consequence of depth sensing on cell proliferation.

As a control, we also assessed the effects of ions used in this experiment on hMSCs. We encapsulated hMSCs in bulk collagen hydrogels made using the same concentration as the collagen core. We then cultured them in a normal growth medium without supplemented ions (COL), 10 mM CaCl_2_-supplemented medium (COL + Ca^2+^) or 10 mM SrCl_2_-supplemented medium (COL + Sr^2+^). The live/dead staining at day 1 showed no obvious difference in hMSCs viability among the group (Figure S5A). At day 5, most hMSCs in all three collagen hydrogel groups were elongated and spread (Figure S5A). Again, F-action/nucleus staining revealed no obvious difference in their morphology (Figure S5B). These results indicated that the presence of 10 mM Ca^2+^ or Sr^2+^ has no significant effects on hMSCs proliferation and spreading.

#### 2.5.2 Human MSCs Differentiation Response

**Figure 6:**
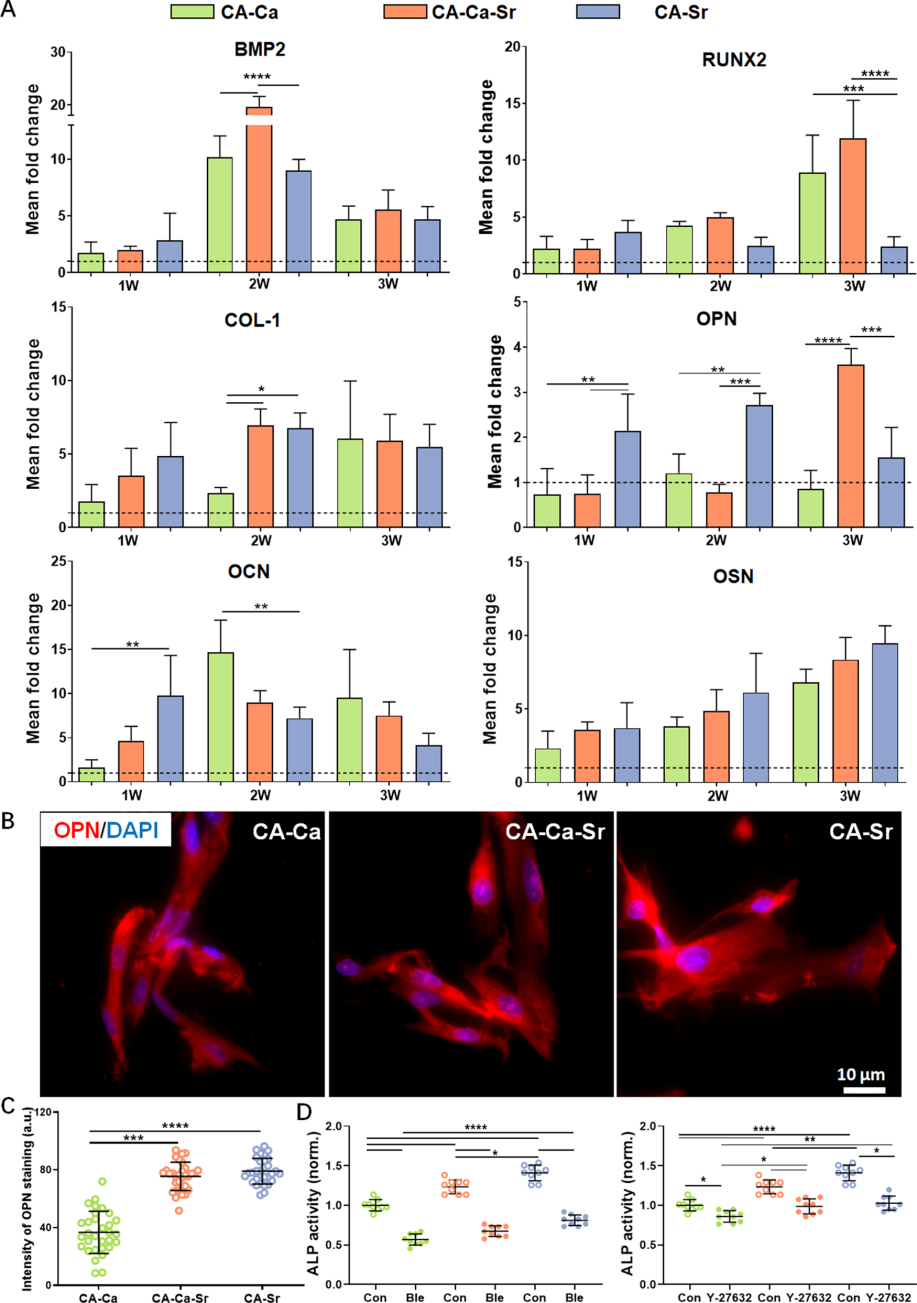
Differentiation of hMSCs in different microfibers. (A) Quantitative osteogenic gene expressions of hMSCs at the end of weeks 1–3 (*n* = 3, biological triplicate). (B) OPN (red) and DAPI (blue) staining of hMSCs cultured in three microfiber groups after 7 days (left) and (C) quantification of the OPN fluorescence intensity (right) (*n* > 20). (D) ALP activity of hMSCs in the presence of blebbistatin (Ble) and Y-27632 supplementary medium after 7 days (*n* = 9, hydrogel samples). *P < 0.05, **P < 0.01, ***P < 0.001, ****P < 0.0001.

After the affirmation of morphological difference, we then assessed the effect of varied, effective stiffness on osteogenic differentiation of hMSCs. A q-PCR (quantitative-PCR) test was adopted to investigate the osteogenic genes expression over a 3-week period. Almost all markers showed a higher expression level for cells in CA–Ca–Sr and CA–Sr fibers than that for cells in CA–Ca fibers at some point during the period (Figure 6A). This suggests stiffer alginate shells promoted osteogenic differentiation of hMSCs.

It is worth noting that the highest expression of each marker occurred at different time points. Because CA–Ca–Sr stiffened with time, its effect did not run concurrently with that of CA–Sr. BMP2 expression was highest in all fibers at week 2 and then reduced at week 3. For RUNX2, the significantly increased levels of RUNX2 expression in CA–Ca and CA–Ca–Sr were observed at week 3, while the highest RUNX2 expression in CA–Sr occurred at week 1 (although there was no significant difference). At week 1, the RUNX2 expression in CA–Sr was higher than CA–Ca–Sr and CA–Ca, as the stiffness of CA–Ca and CA–Ca–Sr was lower than CA–Sr. With increasing time, the soft CA–Ca fiber and dynamic CA–Ca–Sr fiber showed a delayed high expression of RUNX2 (both at week 3), while the RUNX2 expression in CA–Sr decreased with time. This difference in expression time indicates that BMP2 is an early marker for stimulated RUNX2 (the master osteogenic transcription factor) expression.^26^ A significantly higher COL-1 expression was observed in CA–Ca–Sr and CA–Sr fibers at week 2, indicating the active production of a native bone matrix for cells in the stiffening and stiff fibers. The expression levels of OPN and OCN in CA–Ca–Sr and CA–Sr fibers were significantly higher than that in CA–Ca fibers at week 1, after which their expression levels in the CA–Ca and CA–Ca–Sr fibers increased. The expression levels of OSN in CA–Ca–Sr and CA–Sr fibers also appeared higher than that in CA–Ca at week 1, 2, and 3, respectively (albeit with no significance). In the control experiments, no significant differences were observed in the expression levels of alkaline phosphatase (ALP) and OPN gene marker levels over a 3-week period for hMSCs encapsulated in bulk collagen hydrogels with or without ion supplementary medium (Figure S6), suggesting that 10 mM Ca^2+^ or Sr^2+^ alone has no significant effect on osteogenesis differentiation.

Numerous studies have shown a significant increase in OPN mRNA under mechanical forces, as gene encoding OPN protein responds to mechanical stimulation.^26^ OPN proteins are also known to be associated with bone metabolism and remodelling.^27^ Thus, immunofluorescence staining of OPN proteins was further characterized. The intensity of OPN staining in CA–Sr fibers was higher than the other groups (Figure 6B,C), indicating more OPN proteins secreted by hMSCs in a stiff matrix. Both the expression and immunofluorescence studies show stiffening and stiff fibers promote hMSCs osteogenic differentiation during culture.

As mentioned above, the intracellular traction force depends on cytoskeletal contractility. Previous studies show that cells sense the stiffness of the ECM through cytoskeleton contractility, which is often controlled via the Rho/ROCK signal transduction pathway.^28,29^ Cytoskeleton contractility determines the intracellular traction force, which may eventually affect cell fate. To test this, we added the ROCK inhibitor (Y-27632) and the myosin II inhibitor (blebbistatin) into culture medium. The ALP activity results in Figure 6D demonstrate that enhanced osteogenesis in stiff or stiffening fibers involves cytoskeleton contractility. Without inhibitors, the ALP activity in CA–Ca–Sr and CA–Sr fibers was significantly higher than that in CA–Ca fibers, indicating that stiffening and stiff fibers do promote hMSC osteogenic differentiation. After adding inhibitors, the ALP activity within all fibers decreased significantly, and the difference between the three fibers was no longer obvious, indicating intracellular traction force, driven by cytoskeleton contractility, plays an important role in regulating hMSC osteogenic differentiation.

## 3 Conclusions

In summary, we have fabricated stiffening core–shell hydrogel microfibers that provide reliable, dynamic modulation of effective Young’s modulus for cells. The stiffening process of the outer hydrogel layer of microfibers was easily achieved by ion exchange during cell culture and did not affect the properties of the thin, bioactive core. Considering the phenomenon of depth sensing by cells, we calculated the effective Young’s modulus sensed by cells within the static or dynamic fibers. Moreover, cell spreading, osteogenic gene expression, and osteogenic protein expression results confirmed that the effective Young’s modulus within different fibers do have an impact on stem cell fate. This study provides a novel tool for dynamically tuning effective Young’s modulus without changing other biochemical or physical characteristics of cellular microenvironments. This will be conducive to understanding the influence of biomechanical signals on stem cell differentiation and can be utilized to maximize yield of the desirable cell type.

## 4 Experiment Section

### 4.1 Materials

Collagen type I was extracted from the skin of a newborn calf and then purified and dissolved in an acetic acid solution (pH = 2).^30^ Sodium alginate (low viscosity and brown algae), calcium chloride, strontium chloride, sodium hydroxide (NaOH), ascorbic acid, dexamethasone, DAPI (4′,6-diamidino-2-phenylindole), PI (propidium iodide), and tetramethyl rhodamine isocyanate (TRITC)-labeled phalloidin were purchased from Sigma-Aldrich (USA). Calcein-AM and fluorescent microparticles were purchased from Invitrogen (USA).

### 4.2 Fabrication of a Microfluidic Extrusion Device

Fabrication of the microfluidic chip has been described previously.^22^ In brief, the microfluidic chip was made by casting a PDMS elastomer against a silicon master (Figure S7). First, the silicon mold for the chip was fabricated using photolithography.^31^ The width of all side channels was 100 μm, and the width of the middle, main channel, was 300 μm. After treatment of the mold with trichloro(1*H*,1*H*,2*H*,2*H*-perfluorooctyl)-silane in a desiccator under vacuum, Sylgard 184 PDMS mixed in a ratio of 10:1 elastomer/curing agent was poured onto it and then cured in an oven. The cured device was peeled from the mold, connection holes were punched, and then the device was bonded to a glass microscope slide following treatment in an oxygen plasma.

### 4.3 Fabrication of Core–Shell Fibers

Sodium alginate powder was dissolved in deionized water (DI water) to form a homogenous solution. Prior to the fabrication, pH of the collagen solution in 0.5 M acetic acid was adjusted to neutral using a 5 M NaOH solution in an ice bath (to prevent collagen gelation). The neutralized collagen solution of 14 mg/mL was used as the core solution, and the alginate solution of 4 mg/mL in DI water was used as the shell solution. The solutions were drawn into two syringes, connected to the inlets through Tygon tubing (Cole-Parmer, inner diameter: 300 μm, external diameter: 500 μm) and delivered to the chip via two syringe pumps.^22^ The flow rates were fixed at 10 μL/min for alginate solution and 20 μL/min for collagen solution, resulting in a continuous, two-layered hydrogel microfiber. The microfibers were then collected in either 100 mM CaCl_2_ or 100 mM SrCl_2_ solution to set the alginate. The resultant fibers were transferred into a 37 °C incubator to crosslink collagen. Alginate microfibers dissolve in the absence of divalent cations, so for long-term cell culture, either 10 mM CaCl_2_ or 10 mM SrCl_2_ was added to the culture media to maintain the gels. Pure alginate (PA) microfiber, without collagen, was extruded at a speed of 30 μL/min. Pure collagen (PC) microfiber was obtained by dissolving the outer alginate layer of CA–Ca fiber using EDTA. PA and PC are used as the fiber controls.

### 4.4 Morphology Characterization of Two-Layered Microfibers

The dimension of the core–shell microfibers was characterized using a fluorescence microscope with Apotome mode (Zeiss, Germany). To visualize individual layers, red and green fluorescence beads were added to the collagen and alginate solutions at a volume ratio of 0.2%, respectively during the fiber formation. The dimension of the fibers was analyzed using Image J software (NIH, Bethesda, USA). Triplicates per group were tested.

### 4.5 Mechanical Characterization

The tensile modulus of the microfibers was measured using a micro-test tensile device (Deben Microtest, UK) equipped with a 5 N load cell. The compressive Young’s modulus of the microfibers was measured using AFM (NanoWizard II Bio AFM, JPK Instruments) mounted on an inverted optical microscope (Zeiss Observe). Force indentation measurements were carried out using FORT TL (Nanoworld) probes with a nominal spring constant of 0.6–3.7 N/m and a 4.89 μm silica microsphere (Bang Labs) attached. To measure the whole fiber, the fiber was attached to an AFM Petri dish by sealing each end to a Petri dish. To measure the cross-section of fibers, they were embedded in 4% low melting point agarose and sectioned. Each section was then sealed to a Petri dish and was mounted onto a heater attachment stage (JPK Instruments) at 37 °C during the AFM measurements. Force spectroscopy measurements were performed on 50 randomized locations on each sample by applying a 3 nN force indentation.^32^ The Hertzian spherical model was applied to the approach force distance.

### 4.6 Cell Culture and Cell-Laden Microfiber Fabrication

Human osteoblast-like cells (MG63s) were cultured in a Dulbecco’s modified Eagle’s medium (Gibco, USA), with 10% fetal bovine serum (FBS; Gibco, USA) and 1% penicillin/streptomycin (Gibco, USA). hMSCs from bone marrow were maintained in a mesenchymal stem cell growth medium (Promocell, USA). The hMSCs were mixed in the collagen precursors at a density of 2 × 10^6^ cells/mL. Then, a cell-collagen mixed solution and pure alginate solution were delivered into the chip as described before. The collected cell-laden fibers were cultured in medium and the medium was refreshed every 3 days.

### 4.7 Cell Viability and Staining

Cell-laden hydrogel microfibers were taken out and stained with Calcein-AM and PI to investigate the proliferation, distribution, and morphology of cells. For F-actin cytoskeleton staining, the cell-laden microfibers were fixed for 1 h with 4% paraformaldehyde in 0.9% NaCl, permeabilized with 0.1% Triton-X in 0.9% NaCl, and blocked with 1% bovine serum in 0.9% NaCl for 90 min to eliminate nonspecific bindings. The cell-laden microfibers were then incubated with Alexa 488-conjugated phalloidin (5 units/mL, Invitrogen, USA) for overnight at 4 °C and then rinsed with 0.9% NaCl.

For immunostaining, cell-laden microfibers were rinsed in 0.9% NaCl solution, fixed with ice acetone for 15 min. Permeabilization of cells was performed in 0.2% Triton X-100 for 90 min. The fixed cells were blocked with 5% bovine serum albumin (BSA) at room temperature for 90 min and then incubated in the primary antibodies Anti-OPN (5 μg/mL, Mouse IgG, ThermoFisher) in 1% BSA at 4 °C overnight. The fluorescent-tagged secondary antibody (Goat Anti-Mouse IgG-Alexa Fluor-488, 5 μg/mL, ThermoFisher) was added in 1% BSA for 60 min at room temperature. Microfibers were stained in DAPI solution for 5 min and then washed for fluorescence imaging.

### 4.8 ALP Activity

Cell-laden microfibers after 7 days in culture were washed with 0.9% NaCl, soaked in 50 mM EDTA and 0.2 mg/mL collagenase solution in 0.9% NaCl for 10 min at room temperature and then incubated in trypsin solution for 10 min to retrieve cells from the microfibers. The obtained cells were washed in 0.9% NaCl, counted, and lysed for 30 min in a lysis buffer (0.1% Triton X-100, 50 mM Tris-HCl) at 4 °C. The ALP activity was measured with a pNPP Alkaline Phosphatase Assay Kit (Sigma) according to the user’s manual. Bovine ALP (Sigma) was adopted to create a standard curve. Absorbance determined at 405 nm was read by a fluorescent plate reader (BioTek, USA) and normalized to cell counts.

### 4.9 Cytoskeletal Inhibition Studies

Cell-laden microfibers were cultured in the stimulated media with a ROCK inhibitor (10 μM Y27632, Sigma) or with a myosin II inhibitor (50 mM blebbistatin, Sigma) or without any inhibitor (as the control). After 7 days in culture, these cell-laden microfibers were tested for their ALP activity as described above.

### 4.10 Gene Expression

At predetermined time points (1, 2, and 3 week), total RNA was extracted from hMSCs with RNA Isolation Kit (QIAGEN, USA) according to the manufacturer’s instructions. Amplification reactions were performed with a SYBR PrimeScript one-step RT-qPCR from RNA kit (Bio-Rad, USA). The primers that were employed are listed in Table 1. All mRNA expression levels were expressed as threshold cycle (CT) values, and the expression of the housekeeping gene GAPDH was used as an internal control to normalize results. The comparative ΔΔ*Ct* method was used to calculate the relative expression. All samples were analyzed in biological triplicates.

**Table 1:** Primer Sequences for Target Genes

gene	forward primer	reverse primer
**GAPDH**	TCAAGGCTGAGAACGGGAA	TGGGTGGCAGTGATGGCA
**RUNX2**	GGTCAGATGCAGGCGGCCC	TACGTGTGGTAGCGCGTGGC
**BMP2**	CTTCTAGCGTTGCTGCTTCC	AACTCGCTCAGGACCTCGT
**OPN**	AGCTGGATGACCAGAGTGCT	TGAAATTCATGGCTGTGGAA
**OCN**	CAGCGAGGTAGTGAAGAGACC	TCTGGAGTTTATTTGGGAGCAG
**COL-I**	CCATGTGAAATTGTCTCCCA	GGGGCAAGACAGTGATTGAA
**OSN**	AGAATGAGAAGCGCCTGGAG	CTGCCAGTGTACAGGGAAGA

### 4.11 Statistical Analysis

Statistical significance was considered by a one-way or two-way ANOVA test, and levels of *P < 0.05, **P < 0.01, ***P < 0.001, ****P < 0.0001 were determined to be statistically significant between groups.

## Supporting Information Available

Picture of microfibers, fluorescent image of core–shell microfiber laden with different fluorescent particles, Tensile modulus of whole microfiber, Young’s modulus of whole microfiber, divisional Young’s modulus of core–shell microfiber, FE simulation, live/dead staining images, cytoskeleton staining images, and immunofluorescence image (PDF)
