# Supplementary material for: Dynamically Modulated Core–Shell Microfibers to Study the Effect of Depth Sensing of Matrix Stiffness on Stem Cell Fate
Source: ACS Appl Mater Interfaces. 2021 Aug 6;13(32):37997–8006. doi: 10.1021/acsami.1c06752 (PMC8397254; doi:10.1021/acsami.1c06752)
Supplement: Supplementary file 1 — am1c06752_si_001.pdf [file am1c06752_si_001.pdf]

Supporting Information

Dynamically Modulated Core–Shell Microfibers to Study the Effect of Depth Sensing of Matrix Stiffness on Stem Cell Fate

Dan Wei[1,2], Laura Charlton[2], Andrew Glidle[2], Nan Qi[3], Phillip S. Dobson[2], Matthew John Dalby[4], Hongsong Fan[1], Huabing Yin[2]**

[1] National Engineering Research Center for Biomaterials, College of Biomedical Engineering, Sichuan University, Chengdu 610064, Sichuan, China

[2] School of Engineering, University of Glasgow, Glasgow G12 8LT, U.K.

[3] School of Engineering, Institute of Marine Science and Technology, Shandong University, Qingdao 266237, China

[4] Centre for the Cellular Microenvironment, Institute of Molecular, Cell and Systems Biology, College of Medical, Veterinary and Life Sciences, University of Glasgow, Glasgow G12 8QQ, U.K.

*Corresponding author: hsfan@scu.edu.cn, huabing.yin@glasgow.ac.uk

Supplementary Figure S1:

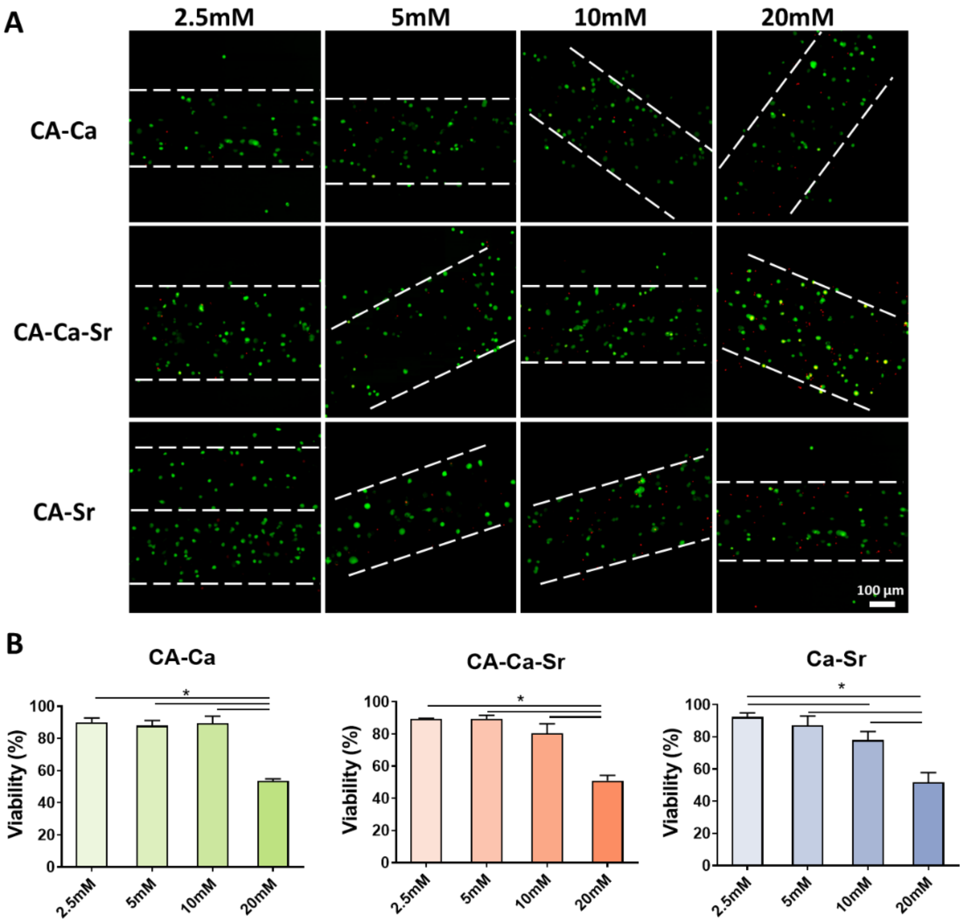

Figure S1. Viability of MG63s in three microfibers cultured in $CaCl_2$ supplemented medium or $SrCl_2$ supplemented medium at various concentrations after 1 day. (A) fluorescence images of live-dead assays. The dotted lie indicates the boundary of the collagen core layer. (B) Percentages of cell viability.

Supplementary Figure S2:

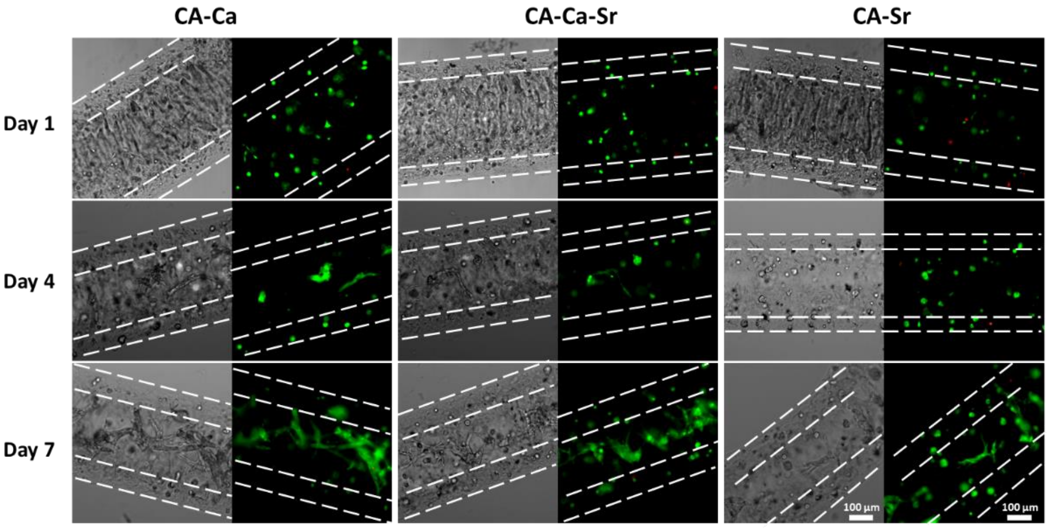

Figure S2. Bright filed images and live/dead staining images of MG63s in three microfibres cultured in 10 mM $CaCl_2$ supplemented medium or 10mM $SrCl_2$ supplemented medium after 7 days.

Supplementary Figure S3:

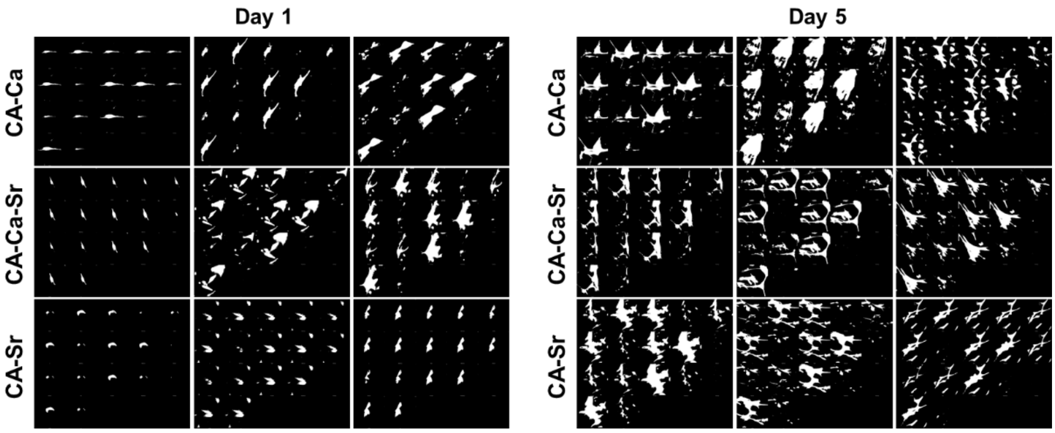

Figure S3. Processed Image J images of hMSCS within three microfibers at day 1 and day 5 used for spreading index analysis.

Supplementary Figure S4:

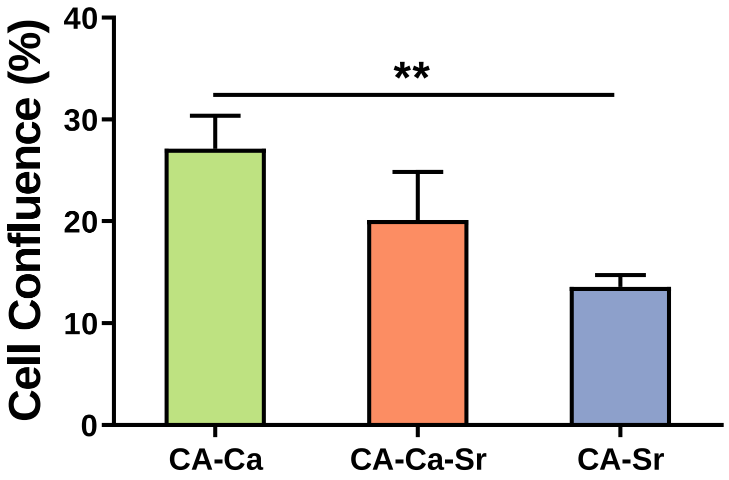

Figure S4. Quantification of cell confluency in three microfiber groups after 10 days.

Supplementary Figure S5:

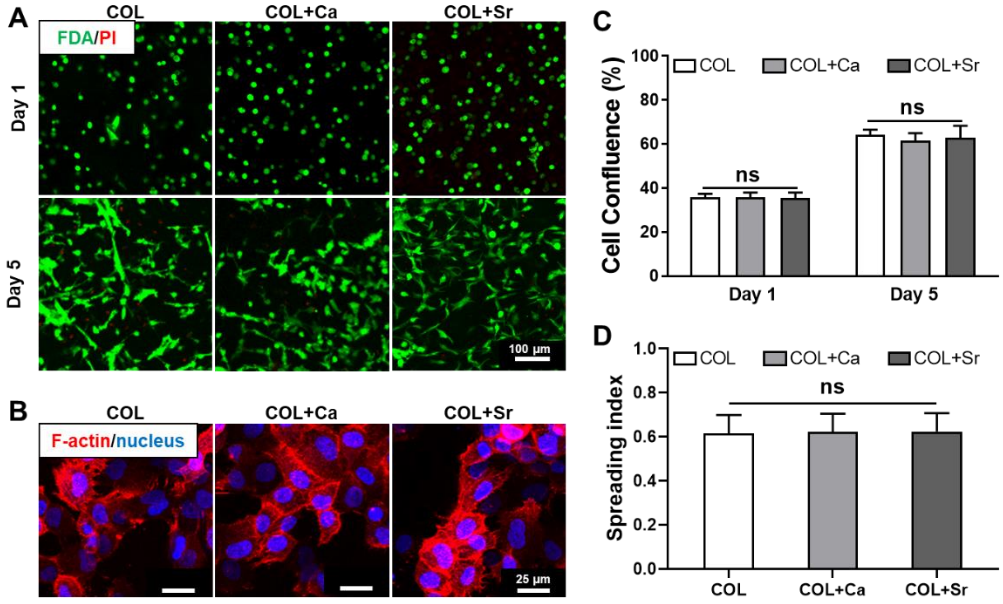

Figure S5. (A) Live (green) /dead (red) staining of hMSCs in bulk collagen hydrogels cultured in normal growth medium without supplemented ions, 10 mM $CaCl_2$ supplemented medium or $SrCl_2$ supplemented medium at day 1 and day 5. (B) F-actin/nucleus staining of hMSCs in bulk collagen hydrogels with different media at day 5. (C) Cell confluence of hMSCs in bulk collagen hydrogels with different media at day1 and day 5. (D) Spreading index of hMSCs in bulk collagen hydrogels with different media at day 5.

Supplementary Figure S6:

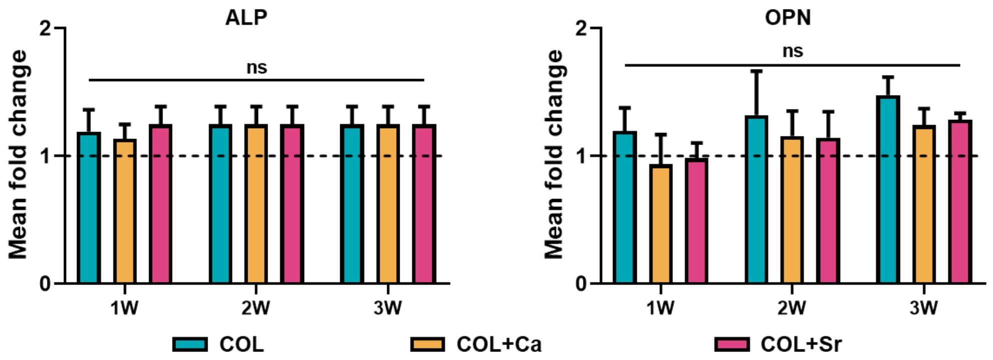

Figure S6. The q-PCR expression levels of ALP and OPN makers of hMSCs in bulk collagen hydrogels in normal growth medium without supplemented ions, 10 mM $CaCl_2$ supplemented medium or $SrCl_2$ supplemented medium at the end of week 1, 2 and 3.

Supplementary Figure S7:

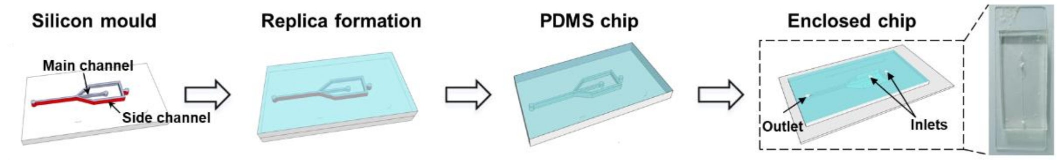

Figure S7. Schematic overview of the fabrication of PDMS chips. The width of the side channels is 100 μm, and the width of the middle, main channel is 300 μm. The depth for all channels is 135 μm.
